# Implementation of Bismuth Chalcogenides as an Efficient Anode: A Journey from Conventional Liquid Electrolyte to an All-Solid-State Li-Ion Battery

**DOI:** 10.3390/molecules25163733

**Published:** 2020-08-15

**Authors:** Rini Singh, Pooja Kumari, Manoj Kumar, Takayuki Ichikawa, Ankur Jain

**Affiliations:** 1 Graduate School of Engineering, Hiroshima University, 1-4-1 Kagamiyama, Higashi-Hiroshima 739-8527, Japan; rini@hiroshima-u.ac.jp (R.S.); tichi@hiroshima-u.ac.jp (T.I.); 2 Department of Physics, Malaviya National Institute of Technology Jaipur, Rajasthan 302017, India; malikpooja720@gmail.com (P.K.); mkumar.phy@mnit.ac.in (M.K.); 3 Natural Science Centre for Basic Research and Development, Hiroshima University, Higashi-Hiroshima 739-8530, Japan

**Keywords:** Bismuth chalcogenides, all-solid-state lithium-ion batteries, electrochemical properties

## Abstract

Bismuth chalcogenide (Bi_2_X_3_; X = sulfur (S), selenium (Se), and tellurium (Te)) materials are considered as promising materials for diverse applications due to their unique properties. Their narrow bandgap, good thermal conductivity, and environmental friendliness make them suitable candidates for thermoelectric applications, photodetector, sensors along with a wide array of energy storage applications. More specifically, their unique layered structure allows them to intercalate Li^+^ ions and further provide conducting channels for transport. This property makes these suitable anodes for Li-ion batteries. However, low conductivity and high-volume expansion cause the poor electrochemical cyclability, thus creating a bottleneck to the implementation of these for practical use. Tremendous endeavors have been devoted towards the enhancement of cyclability of these materials, including nanostructuring and the incorporation of a carbon framework matrix to immobilize the nanostructures to prevent agglomeration. Apart from all these techniques to improve the anode properties of Bi_2_X_3_ materials, a step towards all-solid-state lithium-ion batteries using Bi_2_X_3_-based anodes has also been proven as a key approach for next-generation batteries. This review article highlights the main issues and recent advances associated with Bi_2_X_3_ anodes using both solid and liquid electrolytes.

## 1. Introduction

**Figure 1:**
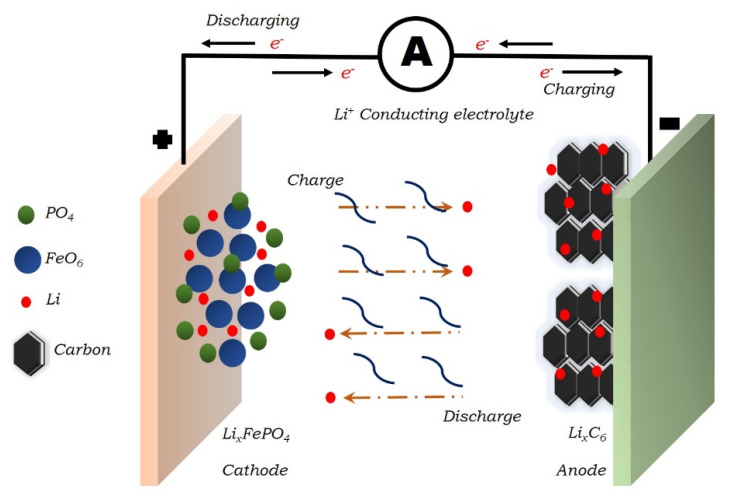
Schematic diagram of Li-ion batteries.

The continuous depletion of fossil fuels and their hazardous byproducts are leading us in the search for clean and sustainable sources of energy [1,2]. During the past few decades, researchers have put huge efforts into the search for renewable sources of energy like solar energy, wind energy, tidal energy, etc. However, their time- and location-dependent availability again forced us to think in the search for energy storage technologies, which can balance the demand and supply efficiently. In addition to this, the reduction in CO_2_ emission is one of the biggest hurdles to overcome the global warming issue. Electrochemical batteries [3,4], fuel cells [5,6,7], solar cells [8,9], etc. are helping to reduce the carbon emission [10,11,12]. The most suitable and advantageous solution to fulfill these requirements is electrochemical batteries. Batteries are the most attractive way to store energy in a chemical form and convert it to electrical energy [13,14,15,16], when needed. The assembly for the battery consists of negative and positive electrodes, whereas electrolytes are used as a separator in the electrochemical batteries. A wide range of classification of batteries is available, including lithium-ion batteries, nickel-cadmium batteries, lead-acid batteries, nickel-metal hybrid batteries, etc. From this available class of batteries, Li-ion batteries are widely used due to their high specific energy density and high cyclic stability in comparison to rest. The schematic diagram of the Li-ion battery is depicted in Figure 1. A Japanese company, Sony, in 1992, launched the first commercial LIBs and it was a huge success, as in the year 1993, it sold 3 million units. Later, in 2001, Tesla commercialized these batteries in the use for electrical vehicle applications. Since then, many communication companies used these batteries in their mobiles, laptops, tablets, etc. These batteries provide many advantageous features on the commercial level i.e., high specific energy, long cycle life, low self-discharge, and their environmental friendliness [10,17].

Continuous advancement in battery technology and smartphones has provided a more user experience. Moreover, these developments continue to demand more cycle life in electronic devices with thinner and lighter Li-ion batteries of higher energy densities. The commercial batteries are developed using liquid electrolytes and graphite-based anodes. However, the graphite has a very low theoretical capacity, i.e., 376 mAhg^−1^. This lower specific capacity led us, researchers, to think of alternative anodes with a higher capacity and long cycle life.

Apart from the issues related to anode materials, the flammable liquid electrolytes associated with the conventional batteries also possess safety issues. These safety issues limit the mass application of this energy storage technique to electrical vehicles. The unsurpassed solution to overcome this problem is the replacement of these liquid electrolytes with solid electrolytes. Additionally, these solid-state electrolytes possess a higher energy density and higher power density. As we are all aware of the fact that organic liquids are more prone to fire, the use of solid electrolytes is a key point for battery technology to improve its safety standards due to the non-use of flammable organic liquid electrolytes [18,19,20,21]. Solid-state electrolytes can provide the advantages of a superior thermal stability, lower flammability, improved durability, and battery design simplicity, over conventional organic liquid electrolytes, even though the room-temperature ionic conductivity in solid electrolytes is still lower than that in liquid electrolytes [22,23,24,25,26,27,28].

The development of alternative anode materials for Li-ion batteries is continuously in progress to satisfy the industrial requirements. Bi and their chalcogenides are promising anodes for Li-ion batteries owing to their high volumetric energy density [29,30,31]. Bismuth (Bi) and its binary chalcogenides (Bi_2_X_3_, X = Te, Se, and S) are known for their various applications like thermoelectric [32,33,34], sensors [35], photodetectors [36,37,38], photoelectrochemical sensors [39,40], etc. due to their unique properties, i.e., their unique layered structure, narrow bandgap, and their environmentally friendly nature [35]. Bi_2_Se_3_ and Bi_2_Te_3_ possess a rhombohedral (R3m) structure, whereas Bi_2_S_3_ consists of an orthorhombic crystal structure. Two Bi layers are sandwiched between X (X = S, Se, Te) layers. These arrangements of Bi and X are called quintuple layers. These layers are connected together by weak Van der Waal forces [41,42]. This gap between the layers provides sufficient space for Li-ion intercalation and further provides channels for the transportation of ions [42]. This feature provides a better efficacy of these materials as an anode for LIBs [35]. The main stumbling block of these materials is their poor cyclability. To solve this issue, researchers have developed many methods to improve their cyclic performance. Nanostructuring is one of the popular methods, which can enhance the electrochemical performance by shortening the Li diffusion path and enhancing the surface area [43,44,45]. Sometimes, the agglomeration of nanoparticles results in the disconnection of active materials from the current collector. To overcome this issue, different forms of carbon matrix were prepared to immobilize the Bi_2_X_3_ nanostructures for the prevention of agglomeration [46,47,48,49,50,51]. In this review, we will discuss the different approaches towards the advancement of electrochemical performances of Bi_2_X_3_ materials.

## 2. Development of Bi-Based Anodes for Li-Ion Batteries

Before understanding the behavior of Bi_2_X_3_ materials, it is imperative to understand the reaction mechanism of Bi towards Li storage. Bismuth has higher theoretical gravimetric and volumetric capacities than carbon-based anodes. Along with the capacity, bismuth has one more advantage of its relatively high potential for Li-Bi alloy formation (0.8 V). This potential prevents the other materials in the anode from participating electrochemically in the reaction. However, its volume expansion is quite high during the alloying reaction, which directly affects the cyclic performance of the battery. In 2001, Crosnier et al. investigated bismuth as an anode material for Li-ion batteries and proposed the following reaction (1):

Bi + Li ↔ Li_3_Bi. (1)

The above-mentioned reaction is reversible. This was the first initiative towards investigating bismuth as an anode material for Li-ion batteries; however, the results of the electrochemical performance were not promising [46,47,48]. Later, in 2002, Xiamming et al. investigated how to reduce volume expansion and the detailed reaction mechanism of Li-Bi reaction [52]. By reducing the thickness of the bismuth anode, they found a better performance in the battery. The thickness of the Bi anode sheet was kept in the micrometers order. The phase diagram of Li-Bi suggests that LiBi and Li_3_Bi can be formed at normal conditions. From cyclic voltammetry (CV) and X-ray diffraction (XRD) studies, two potentials, i.e., 0.830 and 0.805 V, corresponded to the LiBi and Li_3_Bi formation. The authors also suggested that the formation of Li_3_Bi instead of LiBi is mainly responsible for the mechanical degradation of the Li-Bi electrode. The formation of Li_3_Bi alone resulted in a volume expansion of up to 177% when Li was inserted into Bi. The lithium insertion coefficient in LiBi alloy was found to be in the order of 10^−3^ cm^2^/s. This low value suggested the intrinsic properties of the thin-layer electrode. These intrinsic properties include the orientation, crystallographic imperfection, fewer grain boundaries, and porosity of thin-layer electrodes. So, these results were quite interesting to understand the mechanism of alloying in the Li-Bi system. Till now, the reaction mechanism is crystal clear; however, enhancement of the electrochemical performance is still a challenge.

Since the use of nanomaterials in LIBs can improve cyclic stability by shortening the Li-ions’ path, the addition of carbon-based materials with bismuth helps to improve the conductivity of the anode materials. Thus, to improve the performance of the bismuth-based anodes, Park and co-workers investigated the bismuth nanomaterials and their composite anodes [53]. They designed two different samples, i.e., Bi/C (bismuth/carbon) composite and Bi/Al_2_O_3_/C composites. In these samples, Bi nanocrystals were uniformly distributed over the amorphous carbon and mixture of Al_2_O_3_-carbon. Compared to these samples, pure Bi and milled Bi showed a poor performance in terms of cyclability. However, the specific capacity of Bi/C composites after each cycle was found to gradually decrease. The possible reason for this was suggested as the mechanical cracking caused by the large volume change. In the reaction of Li-Bi, the formation of Li_3_Bi causes the major volumetric change as mentioned earlier. Bi/Al_2_O_3_/C electrodes show relatively stable cyclic behavior. The high reversible capacity of 310 mAhg^−1^ and a remarkable retention of 74% was achieved over 100 cycles. The better performance of this composite resulted from the uniform distribution of nanosized Bi crystallites. The strain generated during cycling could be accommodated by the amorphous and inactive Al_2_O_3_ whereas the buffering effect of the amorphous carbon matrix also played a significant role. During charging and discharging, the crystal structure change was observed as follows:

Discharging: Bi (Rhombohedral)→LiBi (Tetragonal)→Li_3_Bi (Cubic).
Charging: Li_3_Bi (Cubic)→LiBi (Tetragonal)→Bi (Rhombohedral).

**Figure 2:**
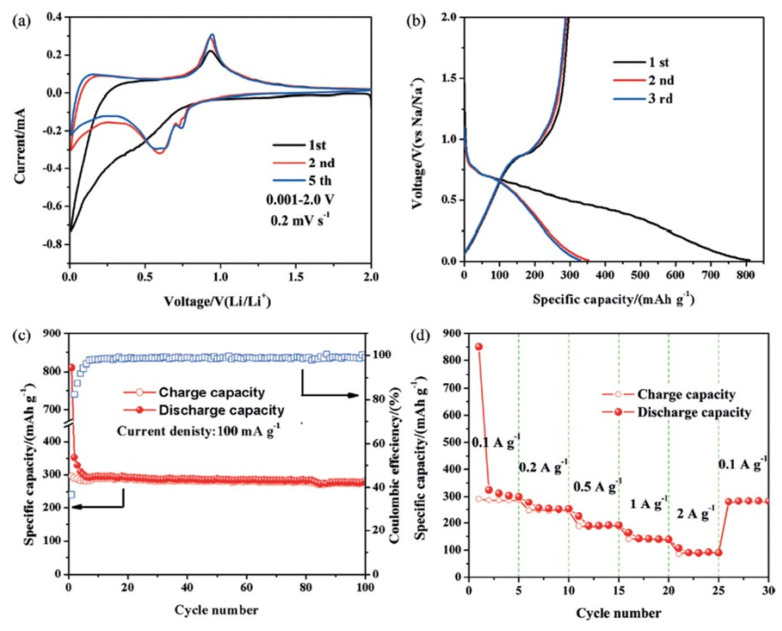
(**a**) Cyclic voltammograms (CV) (**b**) electrochemical charging and discharging (**c**) cyclic stability at 100 mAg^−1^ rates. (**d**) cyclic stability at different current rates of Bi@C microspheres [54]. Reproduced with permission. Copyright 2016, Wiley.

Similarly, Yang and co-workers investigated Bi and Bi encapsulated with carbon (Bi@C) materials for lithium-ion batteries [54]. The reaction mechanism of Bi@C microspheres was understood by the cyclic voltammetry as shown in Figure 2a. No reduction peak was observed in the first cycle of Bi@C samples in the cathodic scan up to 0.01V; however, 2 peaks at 0.75 and 0.63 V originated in the 2nd and 3rd cycles, which were assigned to the LiBi and Li_3_Bi formation, respectively. During the reverse scan, the CV curves showed only one peak instead of two expected peaks, which were suggested due to the overlapping of peaks. The charging/discharging curve (Figure 2b) suggested the first discharge capacity of 810.4 mAhg^−1^, whereas the charging capacity was observed as 276.7 mAhg^−1^. As such, the first coulombic efficiency was found to be only 37%, as depicted in Figure 2c. This low coulombic efficiency was suggested due to solid interface electrolyte (SEI) layer formation, which is quite normal and crucial in the case of liquid electrolytes. The cyclic performance up to 100 cycles was found to be better, i.e., 280 mAhg^−1^ at a current density of 100 mAg^−1^ in the voltage range 0.01–2 V as shown in Figure 2c. Several reports stated that the cyclic stability of Bi-based electrodes remained less than 300 mAhg^−1^ after 100 cycles [55,56,57,58].

**Figure 3:**
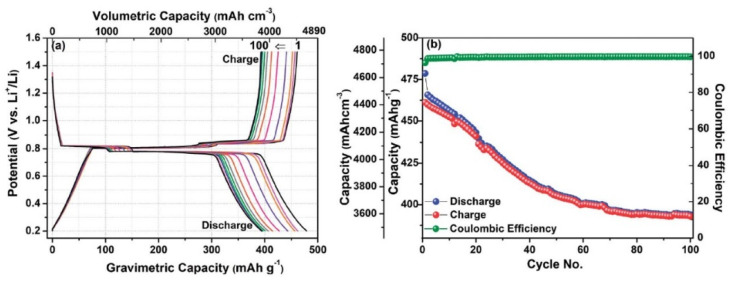
(**a**) Electrochemical charge-discharge of bulk Bi. (**b**) cyclic stability of bulk Bi [59]. Reproduced with permission. Copyright 2018, Royal Society of Chemistry.

These lower capacity and cyclic stability issues were solved by the implementation of Bi anode in all-solid-state Li-ion batteries [59]. Jain and co-workers did excellent work in this regard. Details of all-solid-state Li-ion batteries (ASSLIBs) are already discussed in the introduction section. They proposed the bulk bismuth as a negative electrode for ASSLIBs. In the electrode preparation, lithium borohydride (LiBH_4_) and acetylene black (AB) were milled with bulk bismuth powder. The acetylene black provided the electrical conductivity to the anode whereas LiBH_4_ provided the path to Li ions. The electrochemical performance of the Bi-based electrode was tested between 0.1 and 1.5 V at the current rate of 0.1 C. The discharge capacity decreased from 478.7 to 394 mAhg^−1^ after 100 cycles with stable coulombic efficiency as shown in Figure 3. This battery cell showed good performance as compared to the reports of Bi-based anodes with liquid electrolytes [54,55]. In this study, no cracks were observed even after 100 cycles. This implied that LiBH_4_ and AB worked as a binder, which provided the cushioning effect and accommodated the volume expansion efficiently without breaking the electrode, which resulted in an improvement of the cyclic stability.

## 3. Reaction Mechanism of Bismuth Based Chalcogenides Bi_2_X_3_ (X = S, Se, and Te)

**Figure 4:**
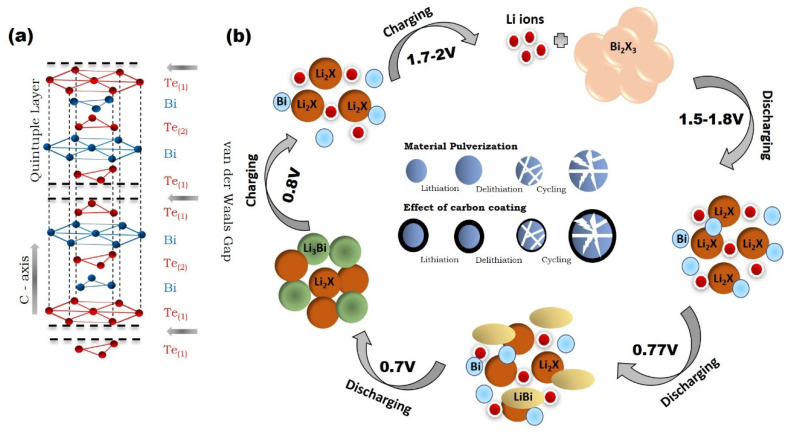
(**a**) Schematic diagram of the crystal structure of Bi_2_X_3_; (**b**) Schematic diagram of the reaction mechanism of the reaction of Bi_2_X_3_ with lithium.

Bismuth chalcogenides have been considered as a promising candidate as anode material for Li-ion batteries due to their unique layered crystal structure. The schematic diagram for layered structure of Bi_2_X_3_ is shown in Figure 4a. During the reaction between Bi_2_X_3_ and Li, a total of 12 lithium react with the Bi_2_X_3_ materials in two steps and the formation of Li_2_X and Li_3_Bi takes place according to the following reactions:

Conversion reaction: Bi_2_X_3_ + 6Li^+^ + 6e^−^ ↔ 3Li_2_X + 2Bi, (2)

Alloying reaction: 2Bi + 6Li^+^ + 6e^−^ ↔ 2Li_3_Bi. (3)

The above reaction and the complete mechanism with plateau voltages during charging and discharging have been explained by many researchers [60,61,62] and can be understood by the schematic diagram as shown in Figure 4b. The first discharge reaction (lithiation) occurs around 1.5–1.7 V depending on element X, where Bi_2_X_3_ converts into Li_2_X and Bi through a conversion reaction. On further discharging, the second reaction takes place at 0.77 V, in which the formation of LiBi takes place, and in the last reaction during discharging, at 0.7 V, the formation of Li_3_Bi occurs. During the charging, a reverse process takes place by the transformation of Li_3_Bi into Bi at 0.8 V in the first step whereas the second step is completed by the conversion of Li_2_X into X at 1.7–2.0 V.

## 4. Bismuth Chalcogenides Materials as an Efficient Anode for Li-Ion Batteries

As mentioned above, all the bismuth chalcogenide materials can accommodate 12 Li ions per mole, which results in the high theoretical capacity of these Bi_2_X_3_ (X = S, Se, and Te), i.e., ~625, ~491, and ~401 mAhg^−1^, respectively. These values of theoretical capacities are quite higher than the commercial graphite-based anodes. Along with the higher theoretical capacity, their unique layered structure, in which the separate quintuple layers are attached with weak Vander Waal forces, provides a suitable gap between layers to easily accommodate the Li ions. This makes these materials suitable for Li-ion storage. In the year 1989, Julien proposed the Li insertion mechanism of Li in the Bi_2_X_3_ matrix [44]. This mechanism was observed and confirmed by electrochemical potential spectroscopy and linear sweep voltammetry techniques. They reported that the lattice of the chalcogenides allows a limited number of Li ions into the matrix, and further lithiation results in the reduction of materials into Li_2_X. A combination of reactions (1) and (2) ensured a huge volumetric expansion, i.e., 230% in the case of Bi_2_S_3_, which is higher than the individual Bi. This volume expansion causes pulverization and cracks even more, and a decay in cyclic stability appeared in the electrochemical performance. Enhancing the cyclic stability of these chalcogenide materials has been considered as a challenge. Among all three Bi_2_X_3_ (X = Te, Se, and S), Bi_2_S_3_ possesses a higher theoretical capacity. However, volume expansion remains an issue.

**Figure 5:**
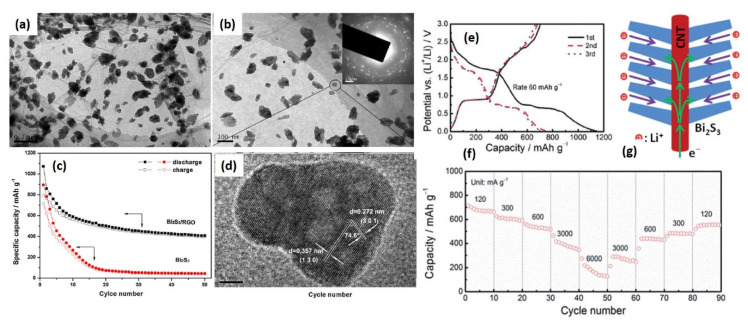
(**a**,**b**) Transmission electron microscopy (TEM) images of anchored Bi2S3 nanoparticles in rGO sheets. (**c**) Cyclic stability of Bi2S3/GO composite and their comparison with bare Bi2S3. (**d**) High-resolution TEM image of Bi2S3 nanoparticle. Reproduced with permission [64], copyright Elsevier 2013. (**e**) Galvanostatic charging-discharging. (**f**) Cycling performance with different current rates of Bi2S3/CNT hybrid structure. (**g**) Schematic representation of branched Bi2S3 and CNT hybrid structures [668]. Reproduced with permission, copyright 2014, Royal Society of Chemistry.

To investigate the electrochemical performance of Bi_2_S_3_, many different approaches have been adopted [60,61,62,63,64]. The most common approach is nanostructuring. Zhang and coworkers synthesized dandelion-like microstructures using the facile reflux synthetic route [65]. This work compared the overall electrochemical performance of nanostructured Bi_2_S_3_ and Bi_2_S_3_-carbon nanotube (CNT) nanocomposites. They observed the first discharge capacity of Bi_2_S_3_ and Bi_2_S_3_/CNT composites as 826 and 574 mAhg^−1^. In the first cycle, the discharge capacity of the composite was found less. However, their coulombic efficiency (63.9%) was found more than that of Bi_2_S_3_ nanostructures (57.8%). The cyclic capacity of Bi_2_S_3_/CNT composite was found to be better than the Bi_2_S_3_, i.e., 247.9 and 74.7 mAhg^−1^ after 50 cycles at 100 mAg^−1^ rates. This improved cyclic capacity was attributed to the improved conductivity and buffering effect provided by the CNT, which compensated the volume expansion associated with the formation of Li_2_S and Li_3_Bi. Further, in their next step, they investigated the performance of Bi_2_S_3_ composites with graphene oxide (GO) [64]. The synthesis of properly anchored Bi_2_S_3_ nanoparticles in graphene oxide sheets was done via the hydrothermal route. The same method was adopted to synthesize Bi_2_S_3_ nanoparticles without the addition of graphene oxide. The transmission electron microscopy (TEM) images of anchored nanoparticles are depicted in Figure 5a,b,d. This helped to better understand the effect of GO on the overall electrochemical performance of Bi_2_S_3_. The size of the nanoparticles was estimated to be 80–100 nm. The anchoring in GO provides some advantages like higher conductivity, efficient accommodation of large volume expansion the same as CNTs, and, additionally, a highly conductive network for rapid electron transport in the electrode during the electrochemical reaction. As evidence of these advantages, the first discharge capacity of the composite was found to be 1073 mAhg^−1^. Further, the cyclic capacity was also found to be comparatively stable, with a capacity of 400.5 mAhg^−1^ at 100 mAg^−1^ rates after 50 cycles. This reversible capacity value was higher than the bare Bi_2_S_3_ nanoparticles and Bi_2_S_3_/CNT nanocomposites. Bare Bi_2_S_3_ nanoparticles showed a cyclic capacity of 44.7 mAhg^−1^, which is less than the dandelion-like Bi_2_S_3_ microstructures. The cycling performance of both the samples are shown in Figure 5c. The possible reason for this was suggested as the agglomeration of nanoparticles during cycling. Additionally, after 10 cycles, the coulombic efficiency of Bi_2_S_3_/GO was found to be 95%, which was attributed to the buffering effect of reduced graphene oxide (rGO) that efficiently mitigates the volume expansion during lithiation and delithiation.

From the above results, it is confirmed that with the anchoring in carbonaceous materials, the electrochemical performance of Bi_2_S_3_ can be improved. In a similar direction with a different approach, Zhao and coworkers did interesting work [66]. They prepared the branched structure of Bi_2_S_3_ with CNT through the sonochemical hydrolysis method. The typical diameter of CNTs was 20–30 nm, and the branched Bi_2_S_3_ nanorods had a 5–10 nm width and 30–80 nm diameter. The schematic representation of branched structures is given in Figure 5g. This hierarchical Bi_2_S_3_-CNT hybrid exhibited a high surface area, flexibility, rich porosity, and direct electron transport pathways. These features provided fast electron and ion transport and structural stability during the cycling process. The initial capacities of the hybrid structure were found to be 1146 and 705 mAhg^−1^, provided the coulombic efficiency of 61.5%. The galvanostatic charging-discharging capacity is shown in Figure 5f. The possible reason for obtaining this efficiency was attributed to the deactivation of the conversion production and formation of the SEI layer. This unique structure provided the short diffusion path for the Li ion to move via Bi_2_S_3_ nanorods. The CNT backbones served the flexible and express path for the rapid charge, which lowered the electrode reaction resistance. As it is previously also mentioned that CNT can accommodate the volume expansion during charging and discharging, here, CNT also provided the higher mechanical strength and also efficiently reduced the mechanical stress and strain during the charging and discharging process. The cyclic capacity of the composite with different current rates is shown in Figure 5f.

Similarly, Jung and co-workers [60] synthesized Bi_2_S_3_ and carbon nanocomposite and further, they compared these materials with bare Bi_2_S_3_. In comparison to the cyclic capacities of both the samples, the capacity of Bi_2_S_3_ faded very rapidly while the Bi_2_S_3_/C nanocomposite electrode showed better performance. The primary reason for the excellent performance was the nanosized effects of Bi_2_S_3_, i.e., large surface area and a reduction in the diffusion length provided better cyclability of the active material. Along with the nanostructuring, an amorphous carbon matrix can accommodate the large volume changes from the active material, while also providing the continuous electric contact network. Further, it acts as a buffer along with the remaining Li_2_S unreacted during the charging step to accommodate the strain generated due to the large volume changes associated with the formation of Li_3_Bi. The Bi_2_S_3_/C anode showed a better performance ca. 500 mAhg^−1^ and 85% capacity retention over the 100 cycles. These results were very promising towards the application of Bi_2_S_3_ as an anode of Li-ion batteries. There are also several other reports on different combinations of carbon-based materials and approaches towards the improvement in the battery performance [67,68,69,70,71,72].

**Figure 6:**
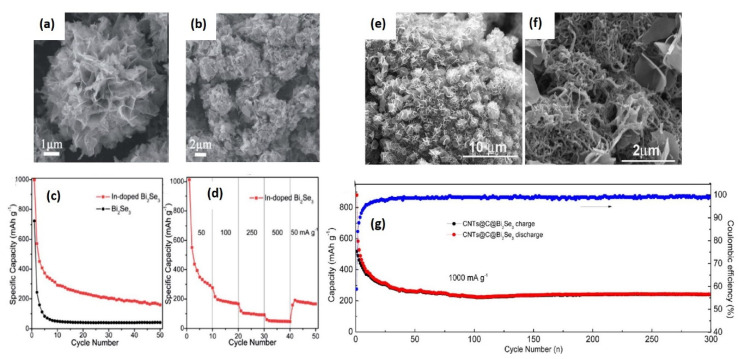
(**a**) Scanning electron microscopy (SEM) image of In-doped Bi_2_Se_3_ nanostructures, (**b**) SEM image of undoped Bi_2_Se_3_ nanostructures, (**c**) cycling performance of doped and undoped Bi_2_Se_3_; (**d**) cycling performance at different current rates, images are reproduced with permission [73], Copyright (2014), Royal Society of Chemistry. (**e**) and (**f**) SEM images of CNTs/C/Bi_2_Se_3_ nanocomposites; (**g**) cycling performance of CNTs/C/Bi_2_Se_3_ composites. Reproduced with permission [77], Copyright (2017), Elsevier.

Similar to Bi_2_S_3_, different approaches towards enhancing the electrochemical performance of Bi_2_Se_3_ were taken into account. To accommodate the volume expansion along with the carbonaceous matrix, metal ion doping was proposed as an attractive way. By taking this approach, Han and co-workers synthesized indium-doped Bi_2_Se_3_ nanostructures through the cation exchange approach [73]. Usually, cation exchange is considered as a versatile approach in nanostructure synthesis. In this approach, the nanostructures’ transformation with appreciable control in the composition, morphology, crystal structure, and doping level can be achieved. In cation exchange, the ionic component of chemical bonds in inorganic materials permits the exchange of one element with the other in the ionic form. They synthesized similar Bi_2_Se_3_ nanostructures to compare the effect of doping on the electrochemical performance. SEM images of In-doped and undoped nanostructures are shown in Figure 6a,b. The first discharge capacity of the doped nanostructures was 997.9 mAhg^−1^ while the undoped Bi_2_Se_3_ showed 723.4 mAhg^−1^. The possible reason for this additional capacity was suggested as a result of the lower charge resistance of In-doped Bi_2_Se_3_, as measured through electrochemical impedance spectroscopy (EIS). The cyclic capacity of doped nanostructures was found to be better than the undoped one as shown in Figure 6c. Doped and undoped Bi_2_Se_3_ nanostructures sustained a reversible capacity of 160.3 and 42.3 mAhg^−1^ after 50 cycles at the 50 mAg^−1^ rate, respectively. Even at different current rates, the cyclic performance was found to be better as depicted in Figure 6d. The better electrochemical performance was achieved due to negligible diffusion through ultrathin nanosheets and faster phase transition. Another possible reason could be the higher surface area, which provided the large contact interface between doped nanostructures and electrolytes. In doping plays a significant role here due to the enhanced carrier density. The cyclic performance of these In-doped Bi_2_Se_3_ nanostructures was higher than Bi_2_Se_3_ nanosheets [74,75], microrods [76], etc.

The Bi_2_Se_3_ with C and CNT composites have been shown to have an improved electrochemical performance compared to the metal ion doping [77]. In this work, Bi_2_Se_3_ nanosheets and their composites with C and CNT were synthesized using the solvothermal technique. SEM images of the synthesized nanocomposites are shown in Figure 6e,f. These composites showed a specific capacity of 432 mAhg^−1^ after 100 cycles at the current density of 100 mAg^−1^. The more interesting point was that even at a high current density, i.e., 2000 mAg^−1^, the specific capacity was maintained at 224 mAhg^−1^. At a higher rate, i.e., 1000 mAg^−1^, composites sustain 242 mAhg^−1^ up to 300 cycles as shown in Figure 6g. In this work, basically, two approaches were combined, i.e., nanostructuring and carbon addition. The advantage of these approaches could be seen in the electrochemical performance. The enhancement in the electrochemical performance was due to the numerous nanocrystals presented in the nanosheets, which caused the shortening of the diffusion path and accommodation of the volume change during electrochemical cycling. Further, the higher surface area of the electrode led to the large contact interfaces between the active material and anode. The additional effect could be seen due to the addition of CNTs. CNTs are flexible conductive networks that accelerate the charge transfer, resulting in the reduction in electrode reaction resistance. It is a known fact that carbon materials consist of high mechanical strength that can also accommodate the stress and strain generation during cycling. These factors are responsible for the excellent electrochemical performance of Bi_2_Se_3_/C/CNT composites.

**Figure 7:**
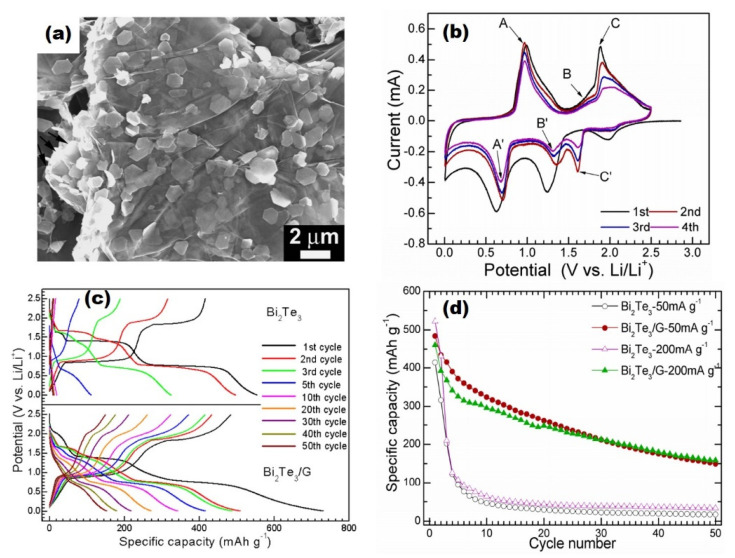
(**a**) SEM image of Bi_2_Te_3_ nanoplates and graphene nanocomposites, (**b**) Cyclic voltammetry at 100 mAg^−1^. (**c**) Galvanostatic charge-discharge performance of bare Bi_2_Te_3_ and Bi_2_Te_3_/G, (**d**) Cycling performance of Bi_2_Te_3_ and Bi_2_Te_3_/graphene composites at two different current rates [78]. Reproduced with permission, copyright 2012, MDPI.

To enhance the cyclic stability of Bi_2_Te_3_, Masood et al. prepared Bi_2_Te_3_ and graphene oxide (GO) composites [31]. The adopted method for synthesis was the polyol method, which signified the distribution of Bi_2_Te_3_ into the GO matrix. The prepared composite showed the first discharge and charge capacity as 752 and 514 mAhg^−1^, respectively, at the current density of 0.1 Ag^−1^. These values were higher than the theoretical capacity of Bi_2_Te_3_ itself, due to the formation of the SEI layer. By incorporating Bi_2_Te_3_ into the GO matrix, the electrical conductivity due to fast Li-ion diffusion was observed to be enhanced. Further, this matrix helped to accommodate the volume changes. The excellent cyclability was found to be 110 mAhg^−1^ in the 500th cycle at the current rate of 0.1 Ag^−1^. These were promising results, which indicated that the GO matrix may help to enhance the cyclability. However, the higher value of the specific capacity was still lacking. Tu et al. prepared Bi_2_Te_3_ nanoplates and graphene nanocomposites to provide a higher electrical conductivity to graphite to enhance the electrochemical performance [78]. Graphene is a monolayer of sp^2^-bonded carbon atoms that possess higher electronic conductivity, excellent mechanical properties, and a higher specific area [79,80,81,82]. These properties make graphene a preferable conducting medium for Li-ion batteries. Bi_2_Te_3_ nanoplates were prepared by the solvothermal method and then composites with Bi_2_Te_3_ nanoplates were prepared for the investigation of electrochemical performance. The SEM of Bi_2_Te_3_ nanoplate/graphene is shown in Figure 7a. The understanding of cyclic voltammetry (CV) is imperative to discuss first. As depicted in Figure 7b, the CV of Bi_2_Te_3_/graphene nanocomposites at 50 mAg^−1^ up to 4 cycles suggested a multi-step reaction of Li with Bi_2_Te_3_. In the first cathodic scan, three different reduction peaks were observed at 2, 1.25, and 0.6 V. These peaks were attributed to lithium insertion, and the formation of Li_2_Te and formation of Li_3_Bi, respectively. The reaction mechanism could be understood by the schematic diagram shown in Figure 4. In the second scan, peaks were observed at 1.65, 1.35, and 0.7 V. The peaks at 1.65 and 1.35 V indicated that the reaction of Te and Li occurred via a two-step reaction with the formation of LiTe_3_ and Li_2_Te. These phases have been reported as stable phases at room temperature [83]. The first discharge capacity of the composite was found to be higher, which was attributed to the formation of a solid electrolyte interface (SEI) layer. The specific capacity of the bare Bi_2_Te_3_ rapidly decayed whereas Bi_2_Te_3_/graphene nanocomposites showed a relatively better performance as shown in Figure 7c. The reason for the stability was proposed based on the restricted crystal growth of Bi_2_Te_3_ due to graphene, resulting in easy diffusion in the Bi_2_Te_3_ matrix. Additionally, separated layers of graphene sheets provided extra vacancies for the Li ion due to the contact sites between the electrolyte. The cyclic performance of the Bi_2_Te_3_/graphene composites was also found to be better than bare Bi_2_Te_3_ as depicted in Figure 7d. After 50 cycles, the charge capacity was obtained as 158 and 33 mAhg^−1^ in case of Bi_2_Te_3_/graphene and bare Bi_2_Te_3_, respectively. The enhanced cyclic capacity was due to the buffering effect of conducting graphene that restrains the aggregation of Bi_2_Te_3_ plates and restricts the volume expansion. Further, this structure also offered the Li-ion diffusion within the free space through the electrode and electrolyte interface. This work was quite important in the direction of the application of Bi_2_Te_3_ and their composites for Li-ion batteries.

## 5. Bismuth Chalcogenides as the Anode in All-Solid-State Lithium-Ion Batteries

**Figure 8:**
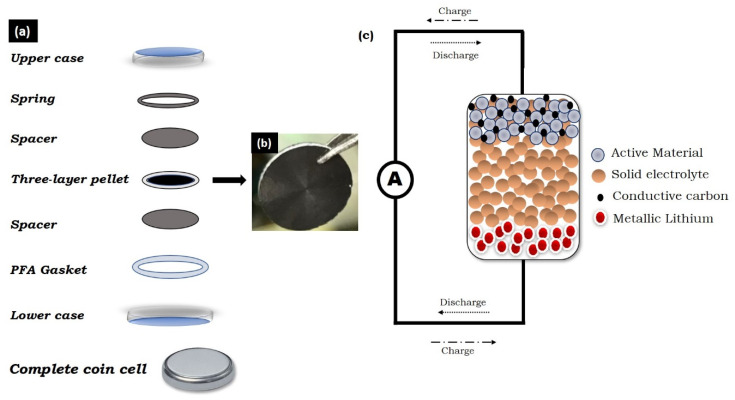
(**a**) Schematic diagram of the cell assembly; (**b**) picture of the actual prepared pellet and (**c**) schematic illustration of the working of the all-solid-state lithium ion battery.

It is already mentioned above that all-solid-state batteries attracted great attention as a next-generation Li-ion battery. Along with the non-flammable behavior of solid electrolytes, they can utilize Li-metal anodes and are able to effectively suppress the formation and growth of Li dendrites. Therefore, the solid-electrolyte strategy provides great opportunities to enhance the safety and energy density of lithium-based batteries [18,84,85,86,87,88]. LiBH_4_ has been considered as a promising solid-state electrolyte in recent years. It consists of an orthorhombic phase at room temperature, which changes to the hexagonal phase at high temperatures (115 °C) [89]. This high-temperature phase shows higher conductivity (10^−3^ Scm^−1^ at 115 °C) than the other sulfide-based electrolytes as well as polymer-based electrolytes. The establishment of this electrolyte for all-solid-state Li-ion batteries has already been reported by several researchers [90,91,92,93]. All the experiments are conducted at a constant current rate, i.e., 0.1 C. The C rate is a measure of the charge and discharge current with respect to its capacity. The 1 C rate delivers the battery’s total capacity in 1 h. A schematic diagram of the all-solid-state Li-ion battery is shown in Figure 8.

In 2018, nanostructured Bi_2_Te_3_ anodes were utilized in Li-ion batteries with lithium borohydride (LiBH_4_) as a solid electrolyte [94]. These nanostructures contained quite a high amount of oxide; thus, the obtained capacity was higher than the theoretical capacity of Bi_2_Te_3_. However, it suffered from poor stability due to the reduction of LiBH_4_ through the reaction with Bi_2_O_3_. Later, to avoid the contribution of oxide in the anode performance, bulk Bi_2_Te_3_, Bi_2_Se_3_, and Bi_2_S_3_ were investigated as anode materials [61,62,95,96]. In these works, LiBH_4_ was used as a solid-state electrolyte. The sample preparation of the anode materials for Li-ion batteries included the 2-h milling of Bi_2_X_3_, acetylene black (AB), and LiBH_4_. The reason for including AB and LiBH_4_ was to enhance the conductivity of the material and to provide the path to Li ions to move with ease. Further, Li foil as a counter electrode, LiBH_4_ as a solid-state electrolyte, and the prepared mixture of the anode was taken to form a three-layered pellet, which was sealed into coin cells for all the electrochemical measurements. The confirmation of separate phases corresponding to LiBH_4_ and Bi_2_X_3_ was done using an X-ray diffraction pattern. This implied that no reaction occurred between Bi_2_X_3_ and LiBH_4_ during milling except the case for Bi_2_S_3_. In the case of the Bi_2_S_3_/LiBH_4_/AB composite, a mechanochemical reaction occurred during milling as well as heating at 120 °C, which converted the initial composite to Li_2_S and Bi phases [61].

Initially, all the electrochemical tests were performed between 0.2 and 2.5 V. The galvanostatic charge-discharge profile is depicted in Figure 6a. Generally, in Bi_2_X_3_ samples, the plateau at around 1.5–1.7 V occurred, corresponding to the movement of Li ions towards the anode from the cathode and the conversion reaction with Bi_2_X_3_ (X = S, Se, and Te), which resulted in the formation of Li_2_X (X = Se and Te) and Bi. As mentioned above, due to the initial reaction of sulfur at the higher temperature, i.e., 120 °C, Bi_2_S_3_ was completely transformed into Bi and Li_2_S, which caused the elimination of the above plateau in the discharge profile of the Bi_2_S_3_ composite. The second and third plateaus in the discharge reaction were observed at 0.75 and 0.71 V, which corresponded to the alloying reaction of Bi and Li and formation of LiBi and Li_3_Bi, respectively [58]. During charging, these plateaus again appeared at 0.82 V, which shows the reversibility of the reaction. The reaction mechanism was established through the CV and XRD results and compiled though the equation mentioned below [94,95,96,97,98]:

Bi_2_X_3_ + 6Li ↔ 2Li_2_X + 2Bi+ Li ↔ 3Li_2_X + Bi + LiBi + 5Li ↔ 3Li_2_X + 2Li_3_Bi. (4)

**Figure 9:**
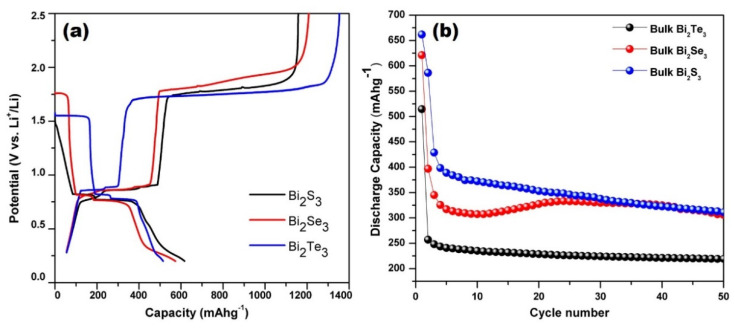
(**a**) First galvanostatic charge-discharge capacity of bulk Bi_2_X_3_ in the voltage range 0.2–2.5 V at the 0.1 C rate; (**b**) Cyclic capacity of Bi_2_X_3_ up to 50 cycles.

In Bi_2_S_3_, the first discharge and charge capacities were found to be 617 and 1145 mAhg^−1^, respectively, as shown in Figure 9a. The discharge capacity was quite similar to the theoretical capacity of Bi_2_S_3_, i.e., 625 mAhg^−1^. The higher value of the charge capacity was proposed due to the thermochemical side reaction between the freshly formed S (as a result of de-lithiation of Li_2_S) and LiBH_4_. This thermochemical reaction caused a poor cyclability and the cell stopped working after only a few cycles. To avoid these thermochemical reactions, the voltage window was restricted from 0.2–1.5 V. The limited potential window helped to achieve a higher cyclic stability and successful operation of the coin cell. After 50 cycles, the discharge capacity was observed to be 311 mAhg^−1^ as shown in Figure 9b, which is quite higher than the Bi_2_S_3_ nanostructures used with liquid electrolytes earlier [57,65]. Similarly, in the Bi_2_Se_3_ sample, the prepared anode showed the first discharge capacity of 621 mAhg^−1^ whereas the charge capacity was found to be 1346 mAhg^−1^. These values were quite higher than the actual theoretical capacity (491 mAhg^−1^) of Bi_2_Se_3_ anodes. The galvanostatic charge-discharge of Bi_2_Se_3_ is also depicted in Figure 9a. In the discharge capacity, the additional capacity was suggested due to the carbon contribution or other side reaction. The higher charging capacity was believed to be due to the same reason as mentioned above for the Bi_2_S_3_ composite. The occurrence of this thermochemical reaction was also confirmed by the noise in the cyclic voltammetry results [61]. This thermochemical reaction hurdled the cycling and the cell stopped working after 10–15 cycles due to the loss of connectivity. The loss of connectivity was confirmed from the SEM micrographs, where the cracks were visible due to these reactions. To eliminate these reactions, the cell potential window was restricted up to 1.5 V, similar to Bi_2_S_3_ composites. The first discharge capacity was found to be 621 mAhg^−1^, which decreased to 306 mAhg^−1^ after the 50th cycle as shown in Figure 9b. Even after restricting the potential window, the cyclic capacity was still quite high in comparison to the Bi_2_Se_3_ nanostructure and their composites reported previously [71,73,74]. After the 50th cycle, no cracks or crumbling were observed in the SEM micrograph. This represented that the restriction of the potential window was advantageous for the elimination of the side reaction and enhancement of the cyclability. A similar behavior was observed for the Bi_2_Te_3_ composite also, where the first discharge and charge capacity was found to be 515 and 1349 mAhg^−1^. After the 50th cycle, the specific discharge capacity was found to be 235 mAhg^−1^ as depicted in Figure 9b [93]. The cyclic capacity of bulk Bi_2_X_3_ after 50 cycles was found to be higher than the reported values for liquid electrolytes [57,65,68,69,72,74,76,94]. A comparison of the values in tabular form is given in Table 1.

The main problem with these Bi_2_X_3_ materials is volume expansion during the electrochemical reaction, which causes a loss in electrical connections during charging and discharging. This problem could be eliminated in all-solid-state batteries. The homogenous distribution of active material throughout the cell, and the addition of AB and LiBH_4_ works as the binder and provides a cushioning effect, thus accommodating the volume expansion easily, which results in an improved cyclic performance.

**Figure 10:**
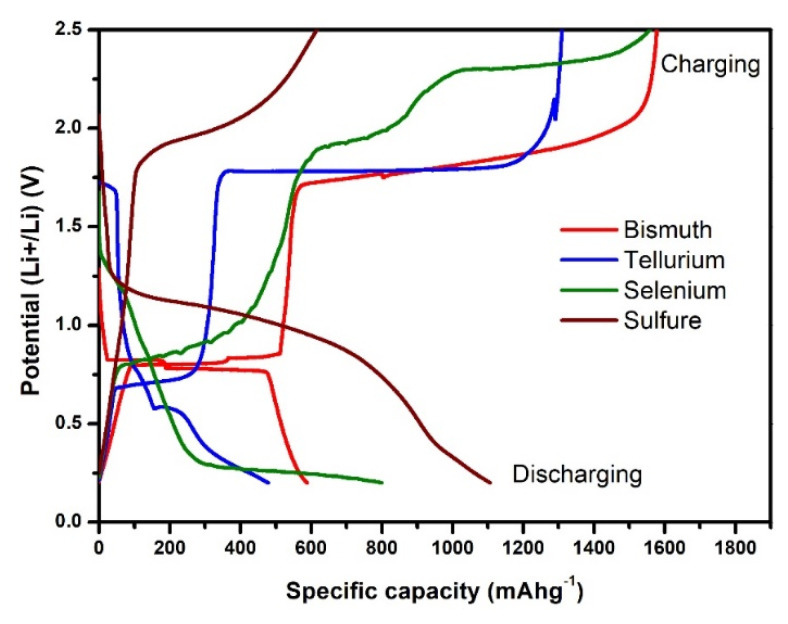
Galvanostatic charging-discharging curve for the first cycle of Bi, S, Se, and Te in the potential window of 0.1–2.5 V.

Previously [61,62,95,96], it was proposed that the long charging plateau around 1.7 V was due to the conversion reaction of Li_2_X to X and the simultaneous thermochemical reaction between freshly formed X and LiBH_4_ presented in the anode layer. This reaction proceeded until LiBH_4_ completely reacted with X materials, which provided excess Li ions. However, no experimental evidence was provided to support our earlier work. Recently, electrochemical charging/discharging was performed for each element, i.e., Bi, S, Se, and Te, as the electrode material. The composites of each element with LiBH_4_ and AB were prepared similarly, and finally, three-layered pellets for each composite were placed in coin cells. The galvanostatic discharge/charge profiles of all four samples are shown in Figure 10. From the figure, it is clear that the reaction plateau of Bi and Te-based composites are at the same position, i.e., 1.7 V. Whereas, the position of the de-lithiation plateau for Li_2_Se and Li_2_S to Se and S is higher, i.e., ~1.9 V. So, it is concluded from this additional investigation that the long plateau above 1.5 V for all the Bi_2_X_3_ composites (except Bi_2_Te_3_) was mainly the result of the thermochemical reaction between readily available Bi (de-lithiation potential from Li_3_Bi to Bi is 0.7–0.8 V) and LiBH_4_, thus forming Li_3_Bi, which immediately de-lithiated into Bi again due to the electrochemical reaction at this potential. This cyclic process continued until the complete consumption of LiBH_4_, which provided an excess amount of Li ions. In the case of Bi_2_Te_3_, Te also contributed to the thermochemical reaction between Te and LiBH_4_. The availability of Te could be possible from the de-lithiation of Li_2_Te at this potential in contrast to the higher potential of delithiation for Li_2_S and Li_2_Se.

To further explore the effect of nanostructuring on the overall electrochemical performance of these materials, Bi_2_X_3_ nanostructures were prepared and electrochemically tested for the all-solid-state Li-ion battery [61]. Bi_2_X_3_ nanostructures were prepared by the hydrothermal synthesis route. Flower-like Bi_2_S_3_ nanostructures were proven to be efficient anodes for all-solid-state Li-ion batteries. The assembly of nanorods having an average diameter of 70 nm and an average length of 300 nm in a flower-like morphology was observed. The main advantage of nanostructuring is the shortening of the Li diffusion path. In the voltage range of 0.2–1.5 V, the first galvanostatic discharge-charge capacities for these Bi_2_S_3_ nanoflower composites were found to be 685 and 494 mAhg^−1^ at the 0.1 C rate. The coulombic efficiency was found to be 95.8%, which is higher than the liquid electrolyte. In comparison with bulk Bi_2_S_3_, the initial discharge capacity of the nanostructures was lower. However, the cyclic stability was found to be better in nano Bi_2_S_3_ composite anodes. After the 50th cycle, the cyclic capacity was observed as 352 mAhg^−1^, which was higher than bulk Bi_2_S_3_. The total decay in capacity was found to be 29.5% in comparison to 47% for bulk Bi_2_S_3_ composites. This nanoflower-like morphology has a higher surface area and excellent charge transfer kinetics due to the reduced diffusion path. These results showed that the electrochemical performance of Bi_2_S_3_ in all-solid-state Li-ion batteries can be improved through nanostructuring. Some similar effects were also observed in Bi_2_Se_3_ and Bi_2_Te_3_ nanostructure composite anode materials. Bi_2_Se_3_ nanostructures in a mixed morphology, i.e., nanoparticles and nanosheets, were prepared by the hydrothermal method [59]. These prepared nanostructures showed a first discharge capacity as 594 mAhg^−1^, which was faded down to 343 mAhg^−1^ in the 50th cycle. The coulombic efficiency till the 50th cycle was maintained at 99%, which is higher than the reported anodes [71,74]. The cyclic stability was found to be better in comparison to bulk Bi_2_Se_3_. After the 50th cycle, the specific capacity for bulk Bi_2_Se_3_ was 306 mAhg^−1^ in comparison to 343 mAhg^−1^ for nanostructured Bi_2_Se_3_. Bi_2_Te_3_ nanorods showed similar effects on the electrochemical performance of composite electrodes [95]. The first discharge capacity of Bi_2_Te_3_ nanorods was observed to be 489 mAhg^−1^, which remained at 235 mAhg^−1^ after the 50th cycle, which is slightly higher than the bulk Bi_2_Te_3_ (219 mAhg^−1^).

## 6. Summary and Future Outlook

In this review paper, we discussed the recent progress of bismuth chalcogenide materials for conventional Li-ion batteries using liquid electrolyte as well as all-solid-state Li-ion batteries. The reaction mechanism of lithium storage in these materials has been well established. These materials have been proven as the superior anodes for lithium-ion batteries with a simple two-step conversion and alloying reaction during lithiation/de-lithiation. The high capacity of 400–625 mAhg^−1^ in comparison to conventional carbon materials attracted researchers worldwide to employ these for practical purposes; however, there are some hurdles to use it as an anode material practically. Large volume expansion is the main issue with bismuth chalcogenide materials. During the past few decades, researchers have put huge efforts into solving these issues through various methods. The first method chosen to overcome these hurdles was nanostructuring. Various nanostructures with different dimensionality were developed and explored for the higher electrochemical performances as compared to the bulk Bi_2_X_3_ materials. Among the several advantages of these nanostructures, the availability of a higher number of active sites and excellent mechanical strength are the main factors that significantly contribute to enhancing the overall performance of Bi_2_X_3_-based anode materials. Carbon-based materials were also used to synthesize the composites to enhance the conductivity and to accommodate the volume expansion during the cycling of these materials.

The excellent approach to switch from the liquid electrolytes to solid-state batteries is a promising way to enhance the performance of overall batteries. A comparison of the electrochemical performance of Bi_2_X_3_ materials in all-solid-state batteries with the conventional Li-ion batteries using liquid electrolytes was made herein. From the obtained results, it was concluded that solid-state batteries are a good alternative for conventional batteries and can be adopted for next-generation Li-ion batteries. The discussed solid-state batteries were mainly composed of LiBH_4_ as a solid-state electrolyte and Bi_2_X_3_/LiBH_4_/AB composites as the electrode. Even though side thermochemical reactions were observed during the operation of these solid-state batteries, the capacity and stability was found to be better for these in comparison to the conventional batteries using liquid electrolytes, e.g., the all-solid-state battery using a Bi_2_S_3_ (bulk)/LiBH_4_/AB composite anode showed a capacity of 311 mAh/g in comparison to < 250 mAh/g for the Bi_2_S_3_/CNT composite in a conventional battery after 50 cycles. Although, LiBH_4_ has a higher conductivity and could be employed for these electrode materials with high stability; however, it has the limitations of a higher working temperature as well as a side thermochemical reaction at a higher potential. These issues lowered the overall capacity in comparison to the theoretical capacity. In the future, it would be interesting to modify LiBH_4_ for its room temperature operation or to explore other solid electrolytes (e.g., sulfide-based electrolytes) with high conductivity at room temperature. The use of sulfide-based electrolytes not only provides a low working temperature, but it may also help to avoid side thermochemical reactions that occur in the case of LiBH_4_.

## Figures and Tables

**Table 1:** Comparitive of discharge and cyclic capacities of different bismuth chalcogenide bulk and nanostructures.

Samples	Method of Synthesis	Electrolyte	1st Discharge Capacity (mAhg^−1^)	Cyclic Stability	Potential (V vs. Li^+^/Li)	Current Density	Ref.
Dandelion like Bi_2_S_3_	Facile reflux method	Liquid	826	74.7 mAhg^−1^ after 50 cycles	0.001–3 V	100 mAg^−1^	[65]
Bi_2_S_3_/CNT composite	Facile reflux method	Liquid	899	247.9 mAh/g after 50 cycles	0.001–3 V	100 mAg^−1^	[65]
Bi_2_S_3_/C composite	Mechanical milling	Liquid	747	500 mAhg^−1^ after 100 cycles	0–2.5 V	100 mAg^−1^	[60]
Bi_2_S_3_	Mechanical milling	Liquid	746	~100 mAhg^−1^ after 25 cycles	0–2.5 V	100 mAg^−1^	[60]
Bulk Bi_2_S_3_	As purchased	Solid	662	311 mAhg^−1^ after 50 cycles	0.2–1.5 V	0.1 C	[61]
Flower-like Bi_2_S_3_ nanostructures	Hydrothermal	Solid	685	375 mAhg^−1^ after 50 cycle	0.2–1.5 V	0.1 C	[61]
Bi_2_Se_3_/graphene	Hydrothermal/Hummers method	Liquid	695	232 mAhg^−1^ after 50 cycle	0.001–3.0	50 mAg^−1^	[97]
Bi_2_Se_3_ microrods	Microwave assisted route	Liquid	870	55 mAhg^−1^ after 50 cycle	0.01–3.0	50 mAg^−1^	[76]
Bi_2_Se_3_ nanosheets/N doped carbon	Solvothermal synthesis	Liquid	942.6	410.6 mAhg^−1^ after 50 cycles	0–3 V	100 mAg^−1^	[98]
Bulk Bi_2_Se_3_	As purchased	Solid	621	306 mAhg^−1^ after 50 cycles	0.1–1.5 V	0.1 C	[62]
Bi_2_Se_3_ nanostructures	Hydrothermal	Solid	594	343 mAhg^−1^ after 50 cycles	0.2–1.5 V	0.1 C	[62]
Bi_2_Te_3_/GO composite	Polyol method	Liquid	752	~110 mAhg^−1^ after 500 cycles	0.01–3 V	0.1 Ag^−1^	[31]
Bi_2_Te_3_ nanoplate/graphene	Solvothermal/Hummers method	Liquid	731	200 mAhg^−1^ after 50 cycles	0.1–2.5 V	50 mAg^−1^	[78]
Bare Bi_2_Te_3_	Solvothermal	Liquid	~560	50 mAhg^−1^ after 50 cycles	0.1–2.5 V	50 mAg^−1^	[78]
Bi_2_Te_3_ nanorods	Hydrothermal	Solid	533	219 mAhg^−1^ after 50 cycles	0.1–1.5 V	0.1 C	[95]
Bulk Bi_2_Te_3_	As purchased	Solid	489	235 mAhg^−1^ after 50 cycles	0.1–2.5 V	0.1 C	[95]
